# Atomic-Scale Imaging of Transformation of Nickel Nanocrystals to Nickel Carbides in Real Time

**DOI:** 10.1021/acsnano.5c06292

**Published:** 2025-06-13

**Authors:** Pu Yan, Dong Zhang, Wendi Zhang, Kaijun Sun, Meng Jin, Thomas W. Chamberlain, Andrei N. Khlobystov, Ute Kaiser, Yuan Hu, Kecheng Cao

**Affiliations:** † School of Physical Science and Technology & Shanghai Key Laboratory of High-Resolution Electron Microscopy, 387433 ShanghaiTech University, Shanghai 201210, China; ‡ School of Chemistry, 4468 University of Leeds, Leeds, West Yorkshire LS2 9JT, U.K.; § School of Chemistry, University of Nottingham, University Park, Nottingham NG7 2RD, U.K.; ∥ Central Facility for Electron Microscopy, Group of Electron Microscopy of Materials Science, Ulm University, 89081 Ulm, Germany

**Keywords:** carbonization, *in situ* TEM, metal nanocrystal, nanocatalyst, carbon nanotube

## Abstract

Transition of metal-to-metal carbide plays a key role in heterogeneous catalysis. We confined nickel nanocrystals in single-walled carbon nanotubes, stimulated delivery of carbon atoms by the 80 keV electron beam, and imaged the entire carbonization process by time-resolved aberration-corrected transmission electron microscopy at the atomic scale. Metal nanocrystal Ni_40_ progressively capturing carbon atoms evolved from pure metal to Ni_40_C_20_ and then to Ni_40_C_40_. The carbonization is accompanied by changes in the structure of the crystal, including a two-dimensionalization process, at the Ni_40_C_40_ stage. This work provides valuable insights into the atomic mechanism of metal carbide formation, which may help to develop stable catalysts and provide a reliable route for synthesizing metal-based two-dimensional materials.

## Graphical abstract

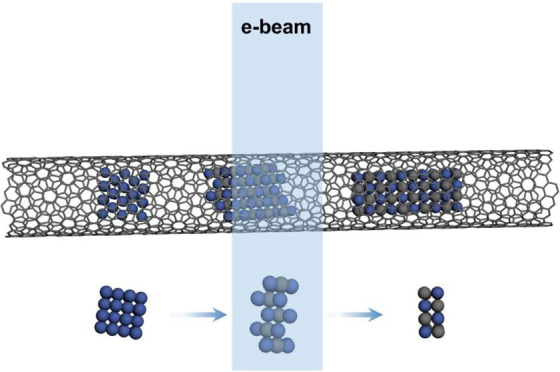

Transition metals, with partially filled *d*-orbital,^1^ allow various types of atoms to be incorporated into the metal lattice, forming compounds with unique physical and chemical properties,^1,2^ such as transition metal carbides,^3,4^ oxides,^5^ and nitrides,^6^ that can be exploited in a wide range of applications in energy storage and conversion,^7^ catalysis,^5,8,9^ biology,^10^ and optoelectronic device.^11^ Nickel, in particular, can be effective as a cocatalyst in the dry reforming of methane^12^ or the copolymerization of olefins.^13^ It is also regarded as a low-cost replacement catalyst for cross-coupling reactions as compared with palladium.^14^ Nickel and other transition metals have also been widely used as catalysts for producing carbon nanotubes (CNT)^15^ and graphene.^16,17^ During catalytic processes, the strong bonding between carbon atoms and transition metal substrate leads to the formation of metastable carbides, which affects the formation rate and growth mode of graphene.^18,19^ For the catalytic growth of CNTs, metallic nickel catalysts exhibit optimal catalytic performance in governing both the growth kinetics and morphological dimensions of CNTs, whereas the formation of interfacial carbide phases inevitably compromises the continuity of catalyst-CNT interfaces during the growth process.^20–23^ Catalyst deactivation is a significant challenge in all of these applications. At high reaction temperatures in carbon-rich environments, the main pathways of catalyst deactivation are related to reactions of metal with carbon (carburization) and particle sintering. Carbon can be chemically adsorbed on metallic catalyst particles in the form of strongly bonded monolayers or physically adsorbed as graphitic multilayers, blocking active sites from the reactants and resulting in catalyst poisoning.^24,25^ An important process is the carbonization of metal particles, leading to the formation of metal carbides,^26^ which have a detrimental effect on the specific surface area, activity, and chemical stability of the catalyst. Reducing the carbonization of nickel in the catalytic process is very important to maintain the activity of the nickel-based catalyst, which requires a thorough understanding of the mechanism of nickel carbonization. Thus, real-time observation of the atomic-scale metal carbonization process would be necessary to provide important guidance for the design of the next generation of stable metal catalysts.

Previous studies have revealed the phase transitions and structure evolution of transition metals during the process of carbonization using *in situ* X-ray diffraction (XRD),^9^ *in situ* X-ray photoelectron spectroscopy (XPS), and *in situ* Raman spectroscopy.^27^ In relevant works, nickel–carbon phase diagram at the nanoscale, carbon solubility in nickel, stability, and wetting properties of nickel carbides have been studied. Among them, DFT calculations have been applied to determine the influence of particle size on the structure and stability of nickel carbides.^20,28–32^ However, the present studies about metal carbonization are mostly focused on the catalyst,^33–36^ and the atomic mechanism for the carbonization process of metal is still unclear.^9,37^ In our previous works, we have systematically studied the interaction between metal nanoparticles and single-walled carbon nanotubes (SWNTs). It has been shown that metal nanoparticles can be entrapped in SWNTs, and that the structural evolution, nucleation behavior, and interactions with carbon can be stimulated and characterized at the atomic level by aberration-corrected transmission electron microscopy (AC-TEM) time-resolved imaging.^38–42^ In this work, we applied this method to nickel nanocrystals for investigating the carbonization process. Our observations of the phase transition, structure evolution, and crystallinity change for nickel nanocrystals shed light on the mechanism of metal carbonization on the atomic scale.

## Results and Discussion

The time series images (Figure 1) show the gradual carbonization process of nickel nanocrystals confined in a SWNT under 80 keV electron beam irradiation (Video S1). The diameter of the host SWNT was measured to be 1.40–1.44 nm, with a chirality of (*m* = 15, *n* = 5) (Figure S2). We constructed the SWNT model and carried out the AC-TEM simulation by QSTEM (Figure S3). The SWNTs in our experiments are measured using a line profile with a diameter of 1.2–1.5 nm (Figure S4). At the initial state (0 s), a nickel nanocrystal is attached to amorphous carbon within the cavity of the host SWNT. The nanocrystal rotates within the nanotube, exhibiting different projections, which were compared with AC-TEM images simulated for a 4 × 5 × 4 nanocrystal of pure nickel, identifying a face-centered cubic (fcc) structure (Figures 2a, S5–S6, and Table S1). There is a 0.3 nm van der Waals gap separating the nickel nanocrystal and SWNT wall, indicating that carbon atoms of the SWNT are not bonded to the nickel nanocrystal at the initial stages of the process between 0 and 20 s (Figure 2b,c). The diameter of SWNT 1.5 nm allows at most four layers of nickel atoms along the *Z* direction, and therefore, the overall 4 × 5 × 4 fcc structure is dictated by confinement in the host SWNT, which limits the maximal total number of nickel atoms in the nanocrystal to 40 (Ni_40_) (Figures 2b,c and S5–S6). A comparison of the experimental and simulated AC-TEM images is shown in Figure 2d,e; corresponding simulation parameters are listed in Table S1. A detailed contrast analysis of the experimental image and correlation with simulations confirm that the observed nanocrystal is a pure nickel metal with a different number of atoms in the atomic columns along the [100] direction (Figure 2d,e).

**Figure 1:**
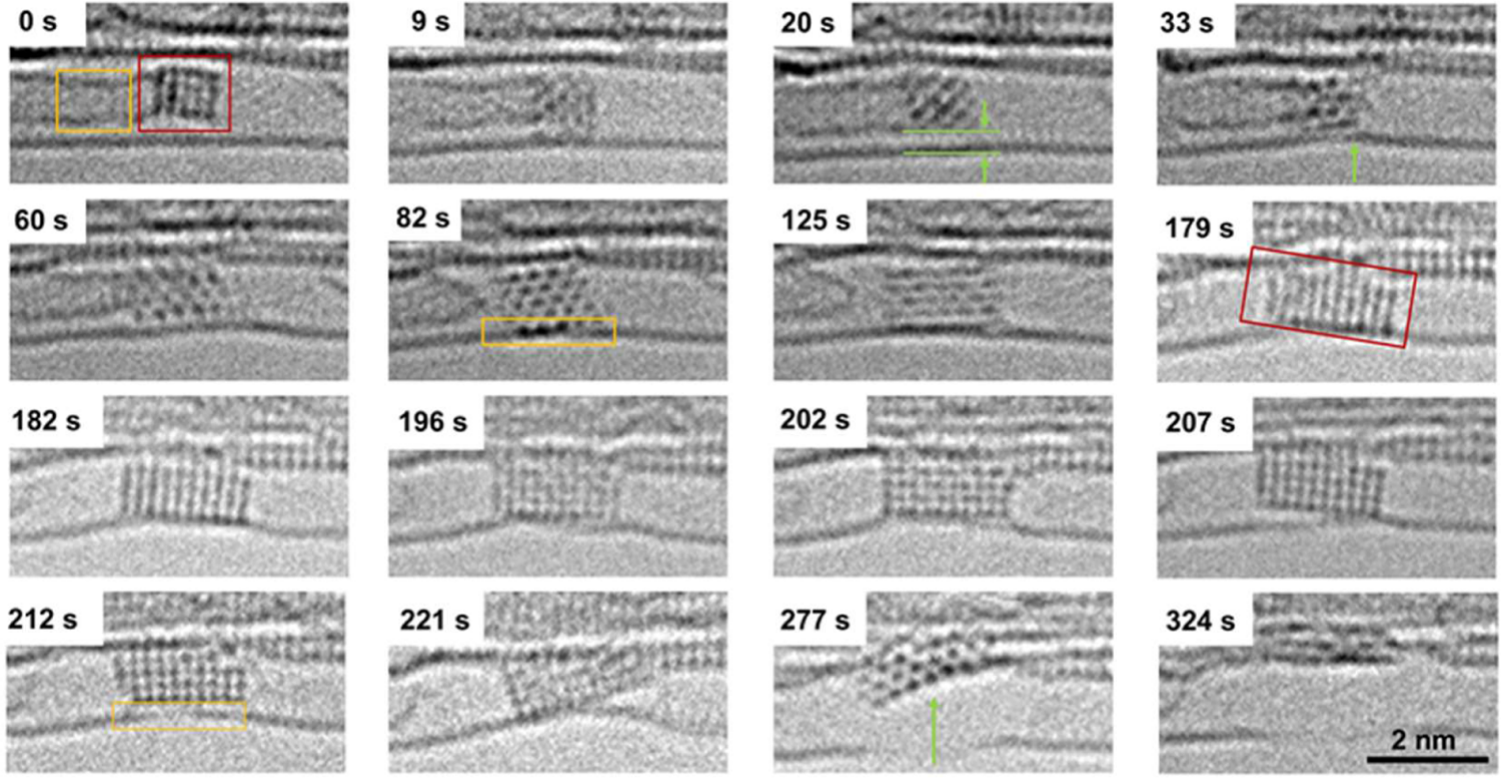
Typical time series of AC-TEM images showing the carbonization process of nickel nanocrystal in the SWNT selected from Video S1 with an exposure time of 1 s per frame. The pure nickel crystal gradually carbonized and cut the SWNT under electron beam irradiation in 324 s (Figure S1). Yellow box in 0 s represents amorphous carbon, and red box represents pure nickel nanocrystal at the start of the process. Green arrow at 20 s represents the van der Waals gap. Yellow box in 82 s represents the bonding between partially carbonized nanocrystals with the outer wall of SWNT. Red box in 179 s represents the two-dimensionalization structure of nanocrystals. The yellow box in 212 s represents the defects of the outer SWNT.

**Figure 2:**
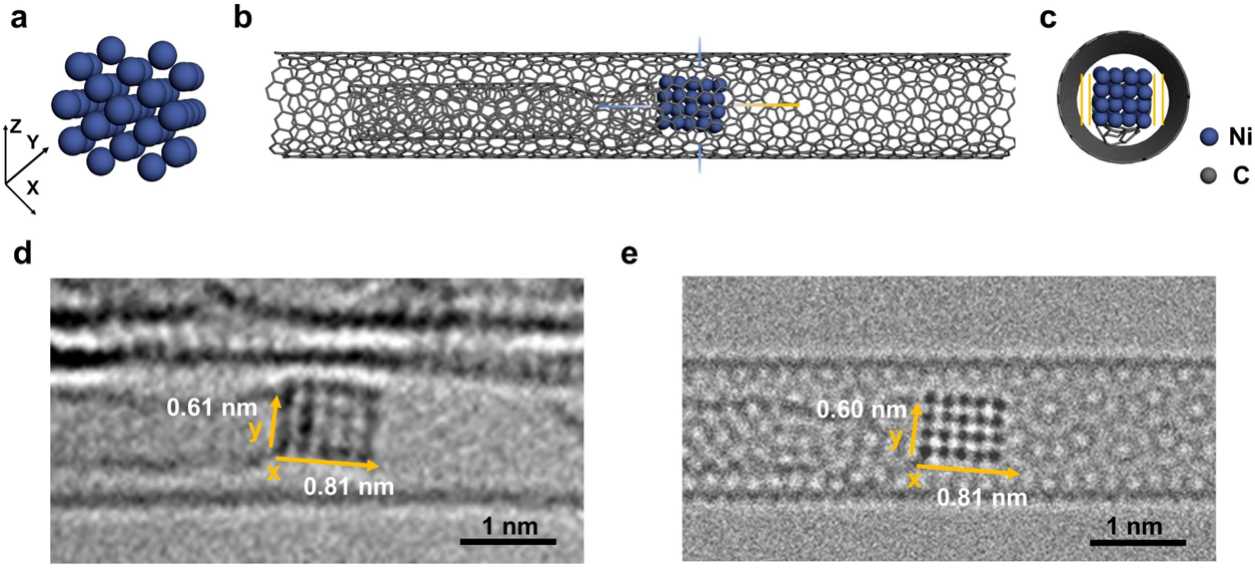
Analysis of the atomic structure of the nickel nanocrystal confined in the SWNT at the initial state (0 s in Figure 1). (a) Structural diagram including 40 nickel atoms at the initial state. (b) The whole model diagram of a nickel nanocrystal in SWNT. (c) End-on view of the model diagram. Yellow lines indicate the van der Waals gap. (d) Experimental image at 0 s and (e) corresponding simulation image based on the model in part (b), view along [100] direction of nickel fcc lattice, distances between rows of nickel atoms are 0.17 and 0.18 nm.

Time-resolved imaging utilizing the 80 keV electron beam both as a probe and stimulus of chemical transformation allowed us to capture the dynamics and reactivity of nickel nanocrystals with atomic resolution in real time. As from 0 to 9 s, the orientation of nickel nanocrystal changes, it begins to interact with the SWNT sidewall, followed by increasing, yet intermittent bonding, from 9 to 33 s with the van der Waals gap opening and closing again (green arrows at 20 and 33 s in Figure 1). At 33 s, a significant deformation of the carbon nanotube due to the strong bonding of the nickel nanocrystal was observed (Figure 1). The nickel nanocrystal noticeably elongated after 60 s, while the Ni–C bond between the amorphous carbon and nickel nanocrystal broke at 82 s. The distances between nickel atoms are increased at this stage, indicating the insertion of carbon atoms into the interstitial spaces of the nickel nanocrystal. The partly carbonized nickel nanocrystal then interacts with carbon atoms of the host SWNT, catalyzing the C–C bond breakage under electron beam irradiation. Unexpectedly, the nickel nanocrystal began flattening from 82 to 207 s, becoming two-dimensional at 179 s while continuing to catalyze the dissociation of carbon atoms on SWNT. At 212 s, the lower part of the SWNT became significantly defective due to the loss of carbon atoms. Most of these lost carbon atoms are activated by the incident electrons and then captured by the carbonizing nickel nanocrystals, while others are ejected into vacuum. At 221 s, the nanocrystal cut into the upper wall of the host SWNT, leading to a phase transition and the formation of a large defect in the carbon nanotube at 277 s due to the carbon loss from the SWNT structure.

Under 80 keV electron beam irradiation, the maximum transferred kinetic energy from an 80 keV electron to carbon and nickel atoms (*E*_T_max__) is 15.8 and 3.23 eV, respectively,^41^ due to a momentum transfer, which is the main driving force for dynamics promoted by the electron beam in atoms and molecules confined inside carbon nanotubes.^43^ The direct displacement threshold energy for atoms in amorphous carbon and SWNT by incident electron impact should be comparable or higher than *E*_T_max__, respectively, and in the region of 17 eV for the nanotube.^44,45^ However, interaction with metal atoms can lower the displacement threshold significantly in the case of nickel, which can be regarded as catalytic C–C bond dissociation.^41^ Thus, it has been proposed that under 80 keV electron beam irradiation, the carbon atoms around the nickel nanocrystal can be activated to become ejected into the vacuum^46^ or captured by the metal nanocrystal and permeate into the lattice forming metal carbide as in the case of Fe,^42^ and in this study, the latter process is being imaged in real time.

There are two types of interstitial spaces in the nickel nanocrystal fcc structure, which, respectively, are octahedral (0.414R_Ni_) and tetrahedral interstices (0.225R_Ni_), with the octahedral interstices being more energetically favorable to host carbon atoms.^47^ Consequently, in the process of carbonization, individual carbon atoms gradually enter into the octahedral interstices of the nickel nanocrystal, which retains the shape but changes the structure in the process. Conveniently, as the nanocrystal is restructuring, the host nanotube allows it to tumble and expose different crystal facets, which helps us to follow the carbonization process (Figures 3–4 and S5).

**Figure 3:**
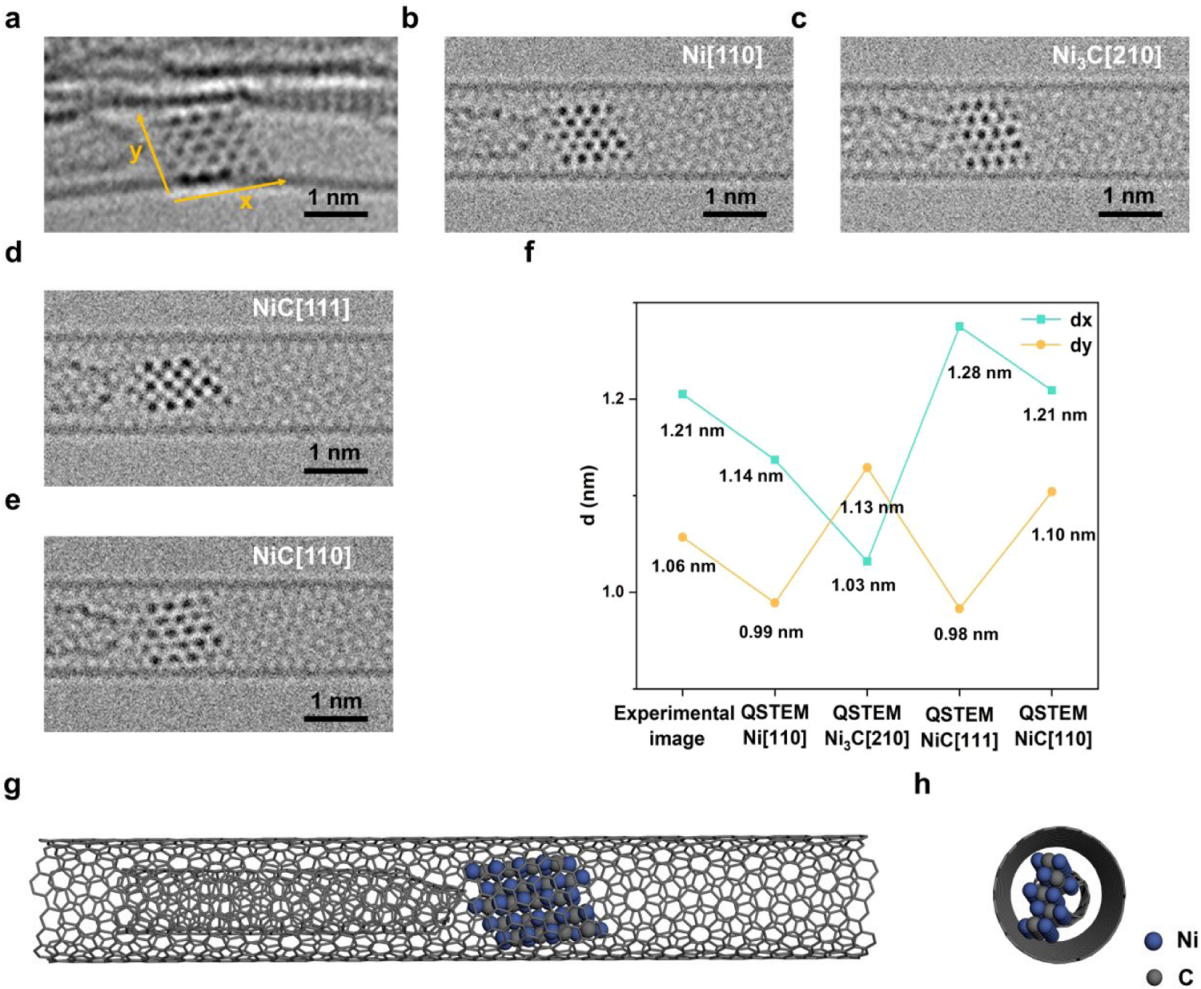
Analysis of the atomic structure of the nickel nanocrystal confined in SWNT at 82 s. (a) Experimental AC-TEM image of the 82 s of the observation process, and the distance between atomic rows of nickel in the *x* direction is 0.32 nm. (b) Simulation image using the nickel metal model viewed in [110] direction, and the distance between atomic rows of nickel in the *x* direction is 0.25 nm. (c) Simulation image using the Ni_3_C model viewed in [210] direction, and the distance between atomic rows of nickel in the *x* direction is 0.22 nm. (d) Simulation image using the NiC model viewed in [111] direction, and the distance between atomic rows of nickel in the *x* direction is 0.29 nm. (e) Simulation image using the NiC model viewed in [110] direction, and the distance between atomic rows of nickel in the *x* direction is 0.32 nm. (f) Size comparison of the real and simulated nanocrystals by measuring their dimensions in the *x* and *y* directions. (g) Model diagram of the partially carbonized nanocrystal of NiC structure (Ni_40_C_20_) in SWNT. (h) Side view of the model in part (g) (relevant model diagram is shown in Figure S7).

**Figure 4:**
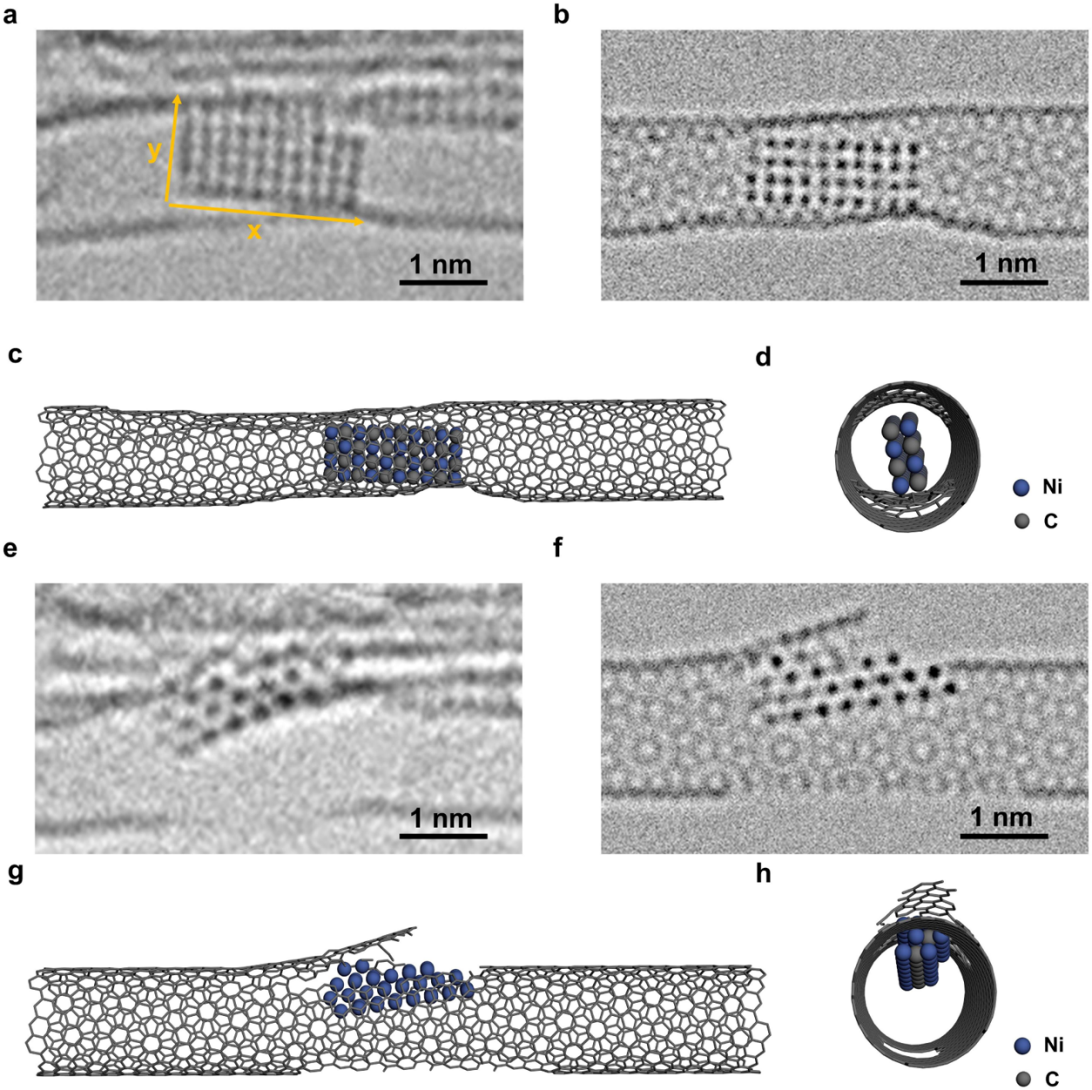
Analysis of the atomic structure of the nickel nanocrystal confined in SWNT at 207 and 277 s. (a) Experimental AC-TEM image of the 207 s from Video S1, and the distance between atomic rows of nickel in the *x* and *y* directions is 0.21 and 0.21 nm, respectively. (b) Simulation image using the NiC model view from [110] direction, and the distance between atomic rows of nickel in the *x* and *y* directions is 0.21 and 0.21 nm, respectively. (c) Model diagram of the fully carbonized nanocrystal in SWNT. (d) Side view of the model in part (c). (e) Experimental image of the 277 s from Video S1. (f) Simulation image of part (e). (g) Model diagram of the nanocrystal cut of the SWNT. (h) Side view of the model in part (g) (relevant model diagram is shown in Figure S8).

For understanding the evolution of the atomic structure of the nanocrystal, we performed meticulous TEM image simulation using atomic models to match the experimental AC-TEM images (Figures 3a–f and S7). Catalyzed by nickel under the 80 keV electron beam, the bonding between the nanocrystal and amorphous carbon dissociated at 82 s. However, at this point, the nickel nanocrystal is clearly in contact with the lower wall of the SWNT. Considering that the atomic ratio of Ni/C in nickel carbide is variable, we chose nickel metal, Ni_3_C, and NiC as the three stable typical structures to build the structural models for TEM image simulation (Figures 3g,h and S7). Because the atomic column contrast of the AC-TEM image is related to the atomic structure and composition of the material, we were able to reveal the nature of the nanocrystal using the contrast information on the experimental AC-TEM images compared to TEM image simulations. Accordingly, the number and position of carbon and nickel atoms in each atomic column were adjusted for the best match of the observed contrast in the experimental AC-TEM images, alongside directions of projection of the model to optimize the match (Figure 3). By measuring and comparing the dimensions of the nanocrystal along the *x* and *y* directions, we were able to estimate the total number of atoms in the nanocrystal of NiC as Ni_40_C_20_, indicating that the nanocrystal had not been completely carbonized. Therefore, the nanocrystal continued to interact and bond with the lower wall of the SWNT. According to TEM image simulations, the carbide structure formed at 82 s changed from the initial four-layered to a three-layered NiC structure as viewed along the [110] direction. By extension of the nickel-based nanocrystal, more octahedral interstices on the surface were exposed.

At 125 s (Figure 1), the partially carbonized nickel nanocrystal disconnected from the amorphous carbon and its lower part bonded strongly to the host SWNT, as the distance between the nanotube sidewall and nanocrystal decreased significantly below the van der Waals gap. This indicates vacancy defect formation in SWNT, which becomes the main source of carbon atoms for the nanocrystal carbonization. From 82 to 179 s, the nanocrystal restructured again, accompanied by enlargement along its *x*-axis and changes to the exposed crystal facet. As at this stage, the nanocrystal is directly bonded to the host SWNT, this prevents its rotation, and therefore the observed changes in the crystal facet must be caused by its lattice restructuring. Furthermore, the interaction between the nanocrystal and the SWNT becomes unstable as the defect develops and the nanocrystal captures more carbon atoms from the SWNT sidewall. At 207 s, the atomic structure of the nanocrystal can be well resolved, which allows quantitative comparison with TEM image simulation (Figures 4a–d). At this point, there are 40 nickel atoms, out of which 20 are positioned in the top layer of the nanocrystal, indicating that the structure becomes double-layered. Compared with the simulation of double-layered Ni_40_ metal (Figures S8–S10), the simulation of the Ni_40_C_40_ nanocrystal is expanded by 14.7% in the *x* direction and 11.5% in the *y* direction. Thus, the nanocrystal reduced its thickness from 4 layers at the start of the process to 3 layers at 82 s and then to 2 layers at 207 s, which can be interpreted as a progressive two-dimensionalization accompanying the carbonization. The two-dimensionalization process may be caused by the stretching force from the carbon atoms on the SWNT to the nickel atoms, or the stretching force is from the flattening of the defective SWNT. In the parallel case presented in Figure S18, from 0 to 123 s, a confined Ni nanoparticle gradually carbonized and extended to a two-dimensional (2D) structure. Then it started cutting the host SWNT from 132 to 194 s. Interestingly, the divided left part of the SWNT dragged the 2D NiC and even pulled out one nickel atom, as pointed out by the arrow in the image of 176 s. Thus, we show that the stretching force between the host SWNT and the formed NiC when the SWNT is breaking is large enough to deform the structure of NiC. This larger stretching force should be the cause of the formation of 2D NiC. This finding provides a new possible mechanism for fabricating 2D materials. To gain a detailed understanding of the atomic structure of the nanocrystal formed at 207 s, we carried out FFT analysis and applied the pure NiC structure for simulation, which has the same geometry as nickel metal but with carbon atoms occupying all octahedral interstices (Figure S11). Our simulation-based NiC model matches well the experimental images (Figures S8–S10). To exclude the influence of the defects of the host SWNT and the defocus changes during the experiment process, we carried out TEM simulation and found that these factors had no effect on the TEM results, as shown in Figures S12 and S13. At 212 s, an extensive defect is formed on the lower part of the SWNT with carbon atoms being extracted and captured by the nanocrystal during NiC formation, leading to the breaking of bonds between the lower part of NiC and the host SWNT. As a result, the tension on the NiC nanocrystal is released such that from 277 s, it became 4-layered again (Figure 4e–h) and began cutting into the upper wall of the SWNT.

In order to verify the reproducibility of the carbonization process of nickel nanocrystals, we presented 9 more cases in Figures 5 and S14–S20. Because the SWNTs we applied to encapsulate Ni nanoparticles have a narrow diameter distribution (1.2 −1.5 nm), the initial Ni nanoparticles also have similar sizes, as shown in all 10 cases including Figure 1. However, the initial Ni nanoparticles in Figures 1,5, and S16 show crystalline structures, while others (in Figures S14, S15, and S17–S20) are amorphous clusters. Under 80 keV electron beam irradiation, all of these 10 Ni nanoparticles obtained carbon atoms from the host SWNTs, gradually carbonized to crystalline NiC nanoparticles. In addition, due to the loss of carbon atoms, the SWNTs became defective and were all eventually cut by the formed NiC nanoparticles (more formed NiC nanoparticles shown in Figure S21).

**Figure 5:**
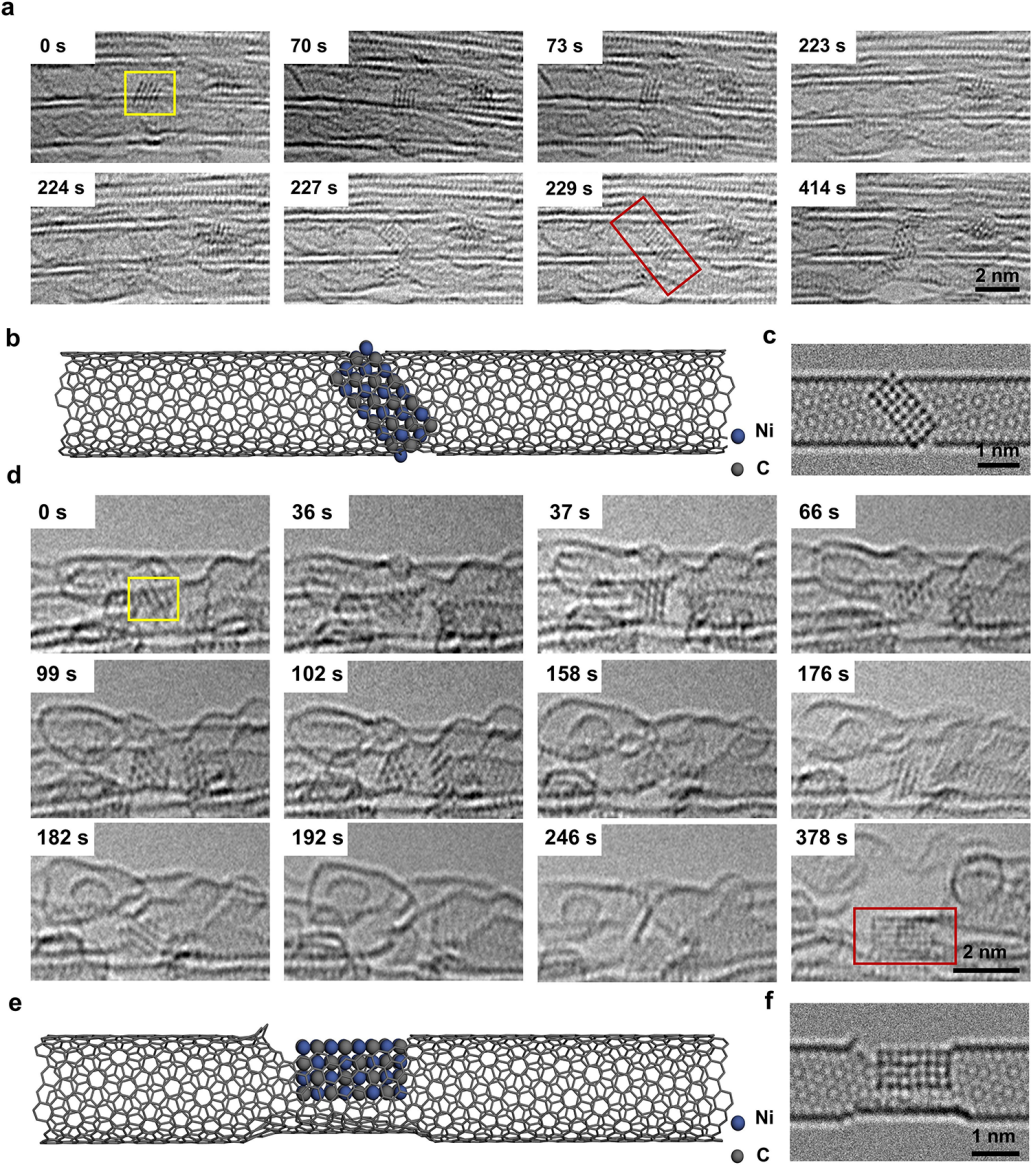
Typical carbonization processes of the nickel nanocrystals in SWNT bundles. (a) Time series AC-TEM images of the carbonization process of a nickel nanocrystal. The nanocrystal in the yellow box is a pure nickel nanocrystal and can also be found in Figure S1. The nanocrystal in red box is a fully carbonized nickel carbide nanocrystal. (b) Model diagram of 229 s in part (a). (c) Corresponding simulation image of the 229 s in part (a). (d) Time series AC-TEM images of the carbonization process of another nickel nanocrystal. The nanocrystal in the yellow box is a pure nickel nanocrystal. The nanocrystal in red box is a fully carbonized nickel carbide nanocrystal. (e) Model diagram of 378 s in part (d). (f) Corresponding simulation image of the 378 s in part (d).

During the entire process in Figure 1, we observed the atomic carbonization process of the 4 × 5 × 4 nickel nanocrystal under the irradiation of 80 keV electron beam. The number of nickel atoms remained constant, as the number of carbon atoms was gradually increasing, leading to Ni_40_C_20_, and it gradually progressed into fully carbonized Ni_40_C_40_. The structure of the nanocrystal undergoes drastic transformations during the carbonization process, including not only lattice reconstitution but also two-dimensionalization as shown in Figure 6. In comparison to conventional thermally induced metal carbonization processes, during which heating increases the mean free energy of atoms in the system, the energy source for the carbonization of nickel in this work is the kinetic energy transferred from the electron beam to the atoms. More details are discussed in the Comparison of the thermally stimulated process and electron beam stimulated process section” in Supporting Information. However, the underlying principles for these kinds of carbonizations are similar, which are based on the fundamental chemical properties of nickel and NiC, as well as the threshold energies for breaking C–C bonds and forming Ni–C bonds. The key advantage of our method is that, as carbon receives significantly more energy from the 80 keV electron beam, C atoms penetrate into interstitial spaces of the nickel nanocrystal, leading to the transformations observed in real time by AC-TEM. Nickel and carbon have rich and versatile chemistry, as prior studies have reported nickel cutting CNTs under 80 keV electron beam irradiation or catalyzing the formation of new carbon structures such as fullerenes.^46,48^ Our current results clearly demonstrate the carbonization of nickel as a competing reaction taking place under the same conditions.

**Figure 6:**
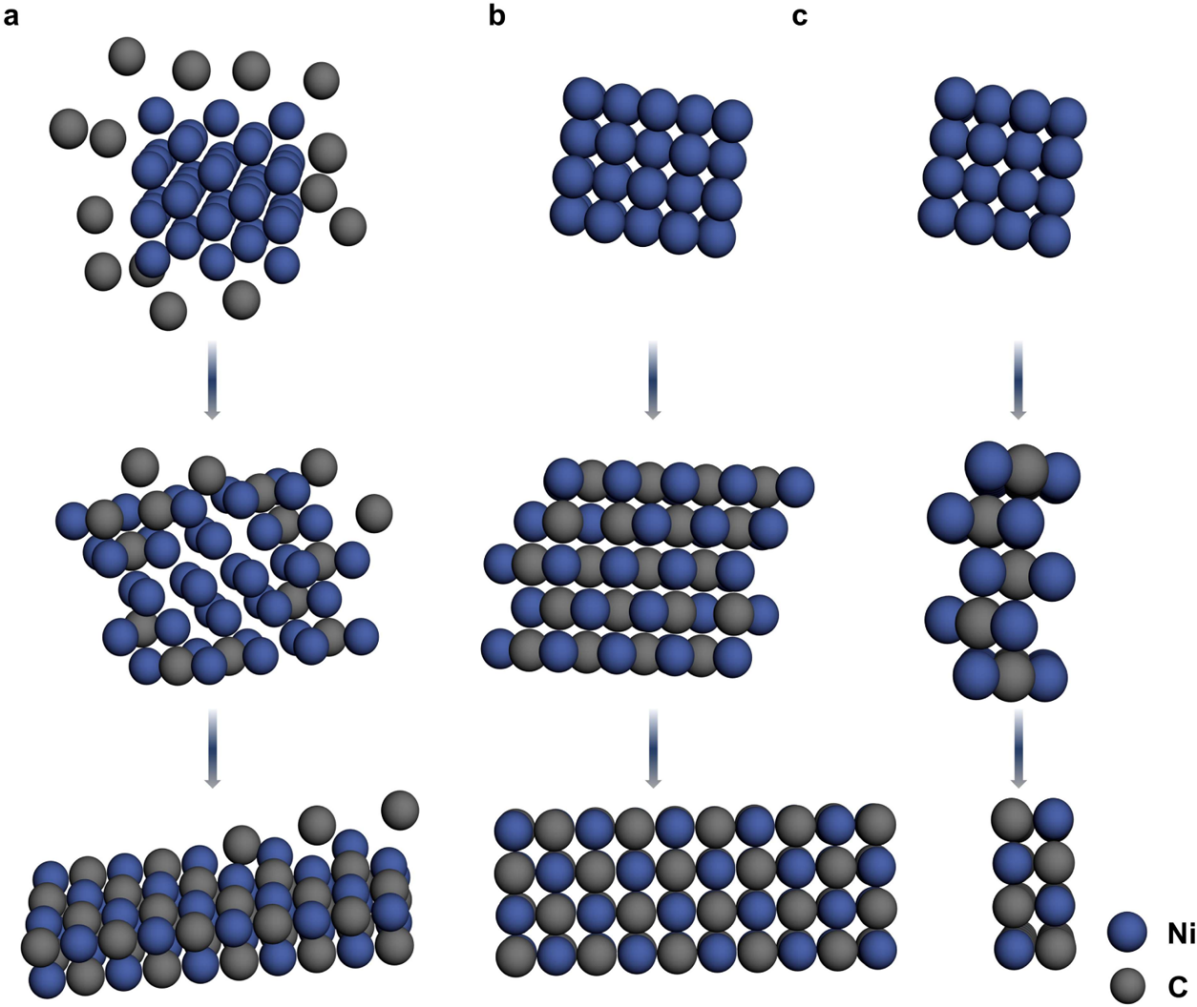
Process map of carbonization of nickel using a model diagram derived from AC-TEM experimental and simulated image analysis. (a) An initial pure nickel nanocrystal immersed in a carbon-rich environment with carbon atoms gradually squeezing into the octahedral interstices of the fcc nickel lattice. (b) Top view of the nanocrystal model diagrams at 0, 82, and 207s. (c) Side view of the model diagram in part (b).

## Conclusions

In this study, we directly imaged the carbonization of nickel nanocrystals confined in SWNTs stimulated by electron beam irradiation in real time and with atomic resolution. The nickel nanocrystal catalyzed the C–C bond breaking and captured the activated carbon atoms from amorphous carbon and the host SWNT under electron beam irradiation. We revealed that the carbonization of nickel is a progressive permeation process of the fcc nickel lattice with carbon atoms, leading to the evolution of pure nickel to Ni_40_C_20_ and then to Ni_40_C_40_. Furthermore, we discovered the two-dimensionalization process during the carbonization of nickel nanoparticles, involving the 4-layered nickel reconstructs to 2-layered NiC, which may be caused by the stretching force from the SWNT sidewall exerted onto the nanocrystal. Thus, utilizing the SWNT as a nano test tube providing a carbon-rich environment for nickel nanocrystals and applying the electron beam of AC-TEM both as an imaging probe as well as a stimulus of reactions, we revealed the atomic mechanism of nickel carbonization. Our findings can provide guidance for designing stable metal-based catalysts as well as new routes toward carbide-based two-dimensional materials.

## Methods

### Sample Preparation

Single-walled carbon nanotubes (SWNTs, Carbon solutions) were thermally treated to open the termini of the SWNTs and to remove the residual amorphous carbon and organic ligand from the outer walls of the SWNTs before use. The as-received SWNTs were heated in air for 30 min at 600 °C with a weight loss of approximately 30% observed for both samples. All other reagents and solvents were used as supplied by Sigma-Aldrich. Nickel­(II) hexafluoroacetylacetonate Ni­(C_5_HF_6_O_2_)_2_ was sealed under vacuum in a quartz ampule and heated at a temperature slightly above its vaporization point (140 °C) for 3 days to ensure complete penetration of the SWNT by Ni­(C_5_HF_6_O_2_)_2_. The sample was cooled to room temperature and washed repeatedly with tetrahydrofuran to remove Ni­(C_5_HF_6_O_2_)_2_ deposited on the outside of the SWNT. The nanotubes filled with Ni­(C_5_HF_6_O_2_)_2_ were then sealed in a quartz ampule under an argon atmosphere and heated at 600 °C, a temperature significantly above the decomposition point of the metal species (∼150–200 °C), for 2 h to decompose Ni­(C_5_HF_6_O_2_)_2_ into the pure nickel metal nanoparticles. Subsequently, C_5_HF_6_O_2_ ligands would thermally decompose into CO_2_ gases and other volatile fragments, which make the internal space in the hosted SWNT more pressurized than the outside. All of the fragments escaped from the SWNT and cannot be observed in TEM. The elementary composition of the prepared sample is determined by EDS mapping as shown in Figure S22.

### Characterization and Simulation

The SWNTs filled with nickel nanoparticles were dispersed in methanol and drop-cast onto lacey carbon–carbon-coated copper TEM grids. Time series AC-TEM images were carried out on an image side C_s_-corrected FEI Titan 80–300 TEM operated at 80 kV at room temperature. HAADF-STEM and corresponding EDS mapping are carried out at a JEOL-Grand Arm 300F. The TEM specimen was heated in air at 150 °C for 5 min shortly before insertion into the TEM column. TEM image simulation was carried out using the multislice program QSTEM. The relevant image simulation is carried out by QSTEM software, and the simulation parameters are listed in Table S1.

## Supplementary Material





## Notes

Additional TEM images of the sample and corresponding simulation images and sample preparation conditions (PDF)
Carbonization of nickel nanocrystal confined in SWNT under electron beam irradiation (Video S1) (AVI)
