# Supplementary material for: Atomic-Scale Imaging of Transformation of Nickel Nanocrystals to Nickel Carbides in Real Time
Source: ACS Nano. 2025 Jun 13;19(25):23306–14. doi: 10.1021/acsnano.5c06292 (PMC12224334; doi:10.1021/acsnano.5c06292)
Supplement: Supplementary file 1 [file nn5c06292_si_001.pdf]

Supporting Information for

Atomic-Scale Imaging of Transformation of Nickel Nanocrystal to Nickel Carbides in Real Time

Pu Yan[1], Dong Zhang[1], Wendi Zhang[1], Kaijun Sun[1], Meng Jin[1], Thomas Chamberlain[2], Andrei N. Khlobystov[3], Ute Kaiser[4*], Yuan Hu[1*], Kecheng Cao[1*]*

Affiliations:

1. School of Physical Science and Technology & Shanghai Key Laboratory of High-resolution Electron Microscopy, ShanghaiTech University, Shanghai 201210, China

2. School of Chemistry, University of Leeds, Leeds West Yorkshire LS2 9JT, UK

3. School of Chemistry, University of Nottingham, University Park, Nottingham NG7 2RD, UK

4. Central Facility for Electron Microscopy, Group of Electron Microscopy of Materials Science, Ulm University, Ulm, Germany

*Correspondence to: caokch@shanghaitech.edu.cn (C.K.); huyuan@shanghaitech.edu.cn (H.Y.); Andrei.Khlobystov@nottingham.ac.uk (A.N.K.) ute.kaiser@uni-ulm.de (U.K)

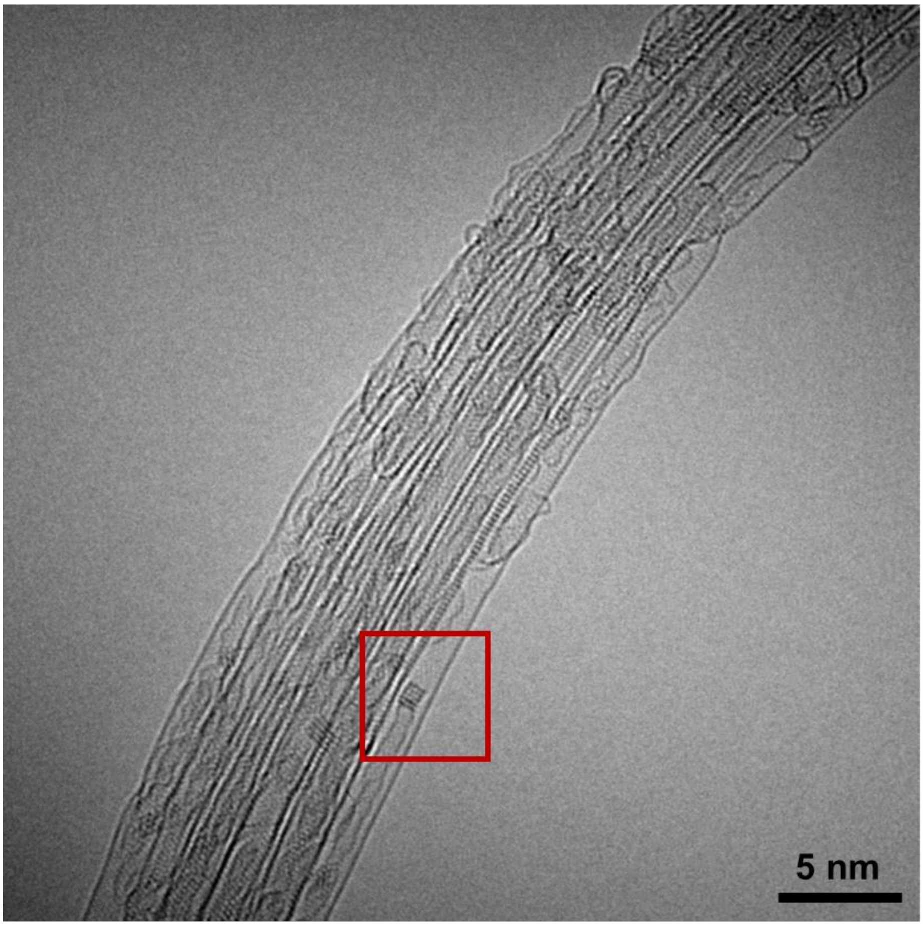

Figure S1. AC-TEM image of nickel confined in SWNT (0 s image of Video S1) showcasing a wide field of view, revealing the extended surroundings of the nickel nanocrystal undergoing gradual carbonization.

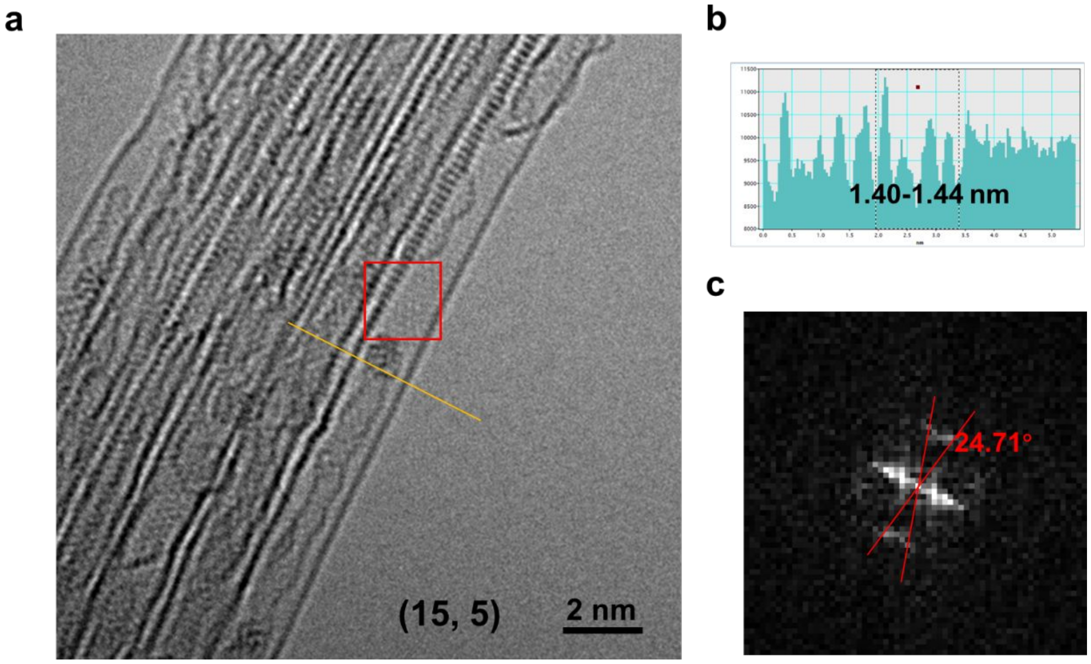

Figure S2. (a) Selected clear frame in Video S1 to determine the charity of this SWNT. (b) Line profile of the orange line in (a) to measure the SWNT diameter. (c)Fast Fourier transformation (FFT) of the red area boxed in (a) for the SWNT. The chiral index of the outer SWNT is determined by measuring the diameter of the SWNT (1.40-1.44 nm) and the characteristic FFT signal in (c). The chiral index of the SWNT is n = 15 and m = 5.

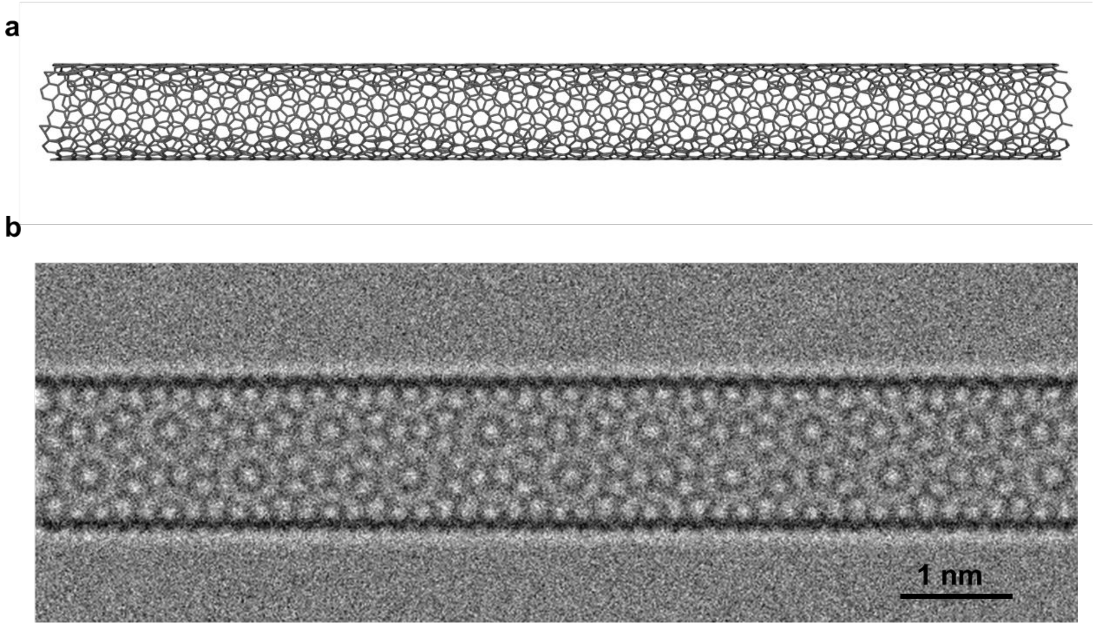

Figure S3. (a) The (15,5) SWNT structural model diagram with determined chiral index of (n=15; m=5). (b)The relevant QSTEM simulation image of (a).

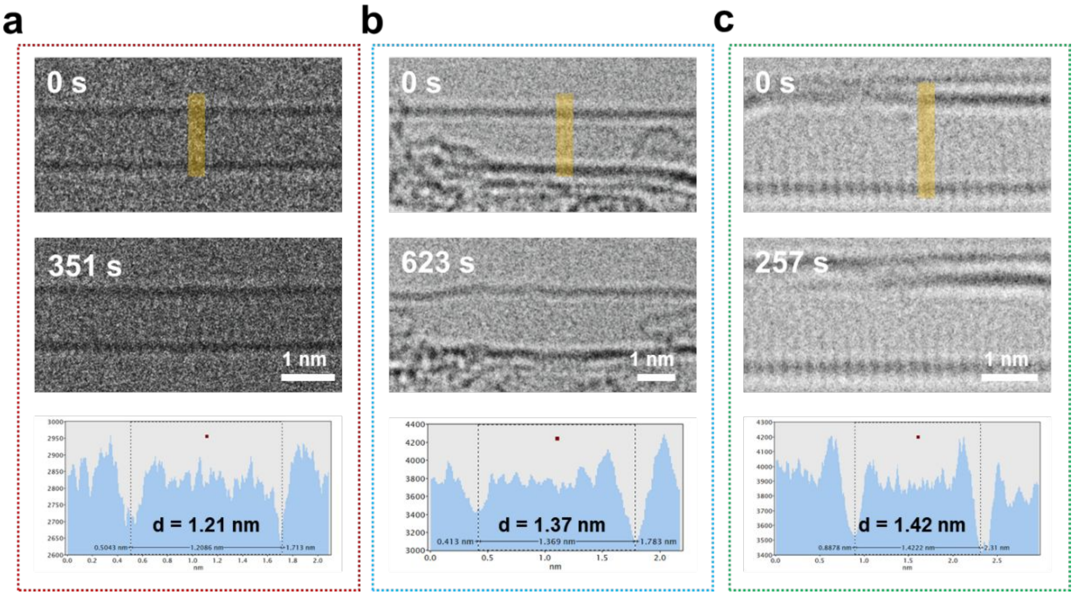

Figure S4. Verification the structural stability of the SWNTs with different diameter under 80 keV electron beam irradiation. (a) TEM images of a SWNT with diameter of 1.21 nm and corresponding line profile. (b) TEM images of a SWNT with diameter of 1.37 nm and corresponding line profile. (c) TEM images of a SWNT with diameter of 1.42 nm and corresponding line profile.

The commercial SWNTs used in this study has narrow diameter range from1.2 to1.5 nm, that have sufficient structural stability under continuous 80 kV electron beam irradiation for up to 10 minutes

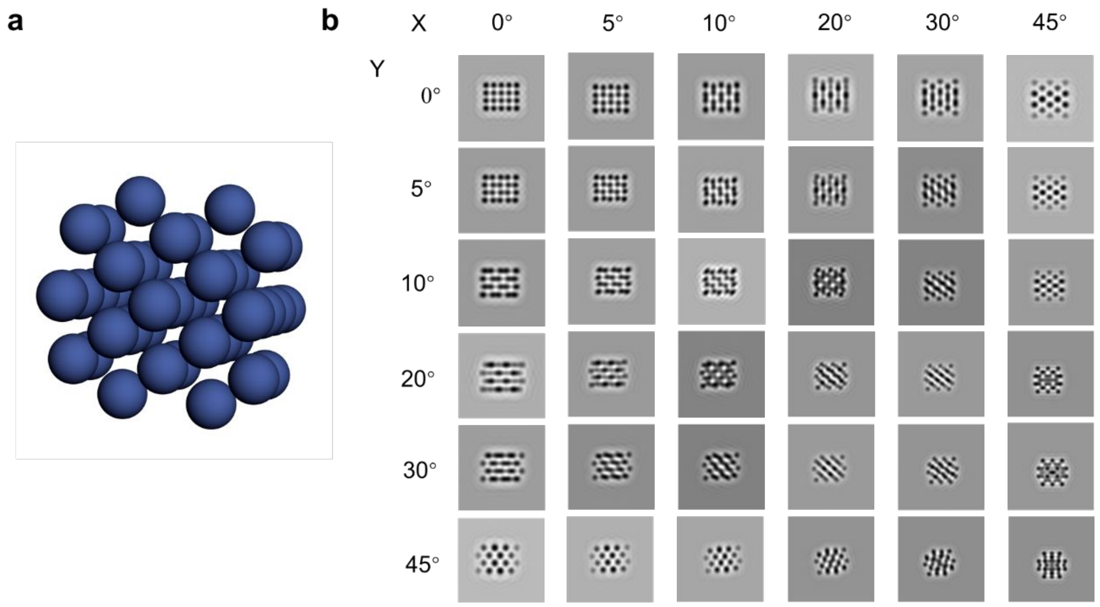

Figure S5. The initial nickel nanoparticle structure of 4×5×4 totally 40 nickel atoms and the simulation images of the nickel model sequential tilt along X and Y direction.

In our study, we conducted rotations of a nickel nanoparticle with initial dimensions of 4×5×4 in the X and Y directions to simulate and confirm that the behavior of nickel nanocrystal within SWCNTs extends beyond simple rotational motion of their own structure.[1-2] Our findings demonstrate further transformations occurring within the nickel nanocrystal.

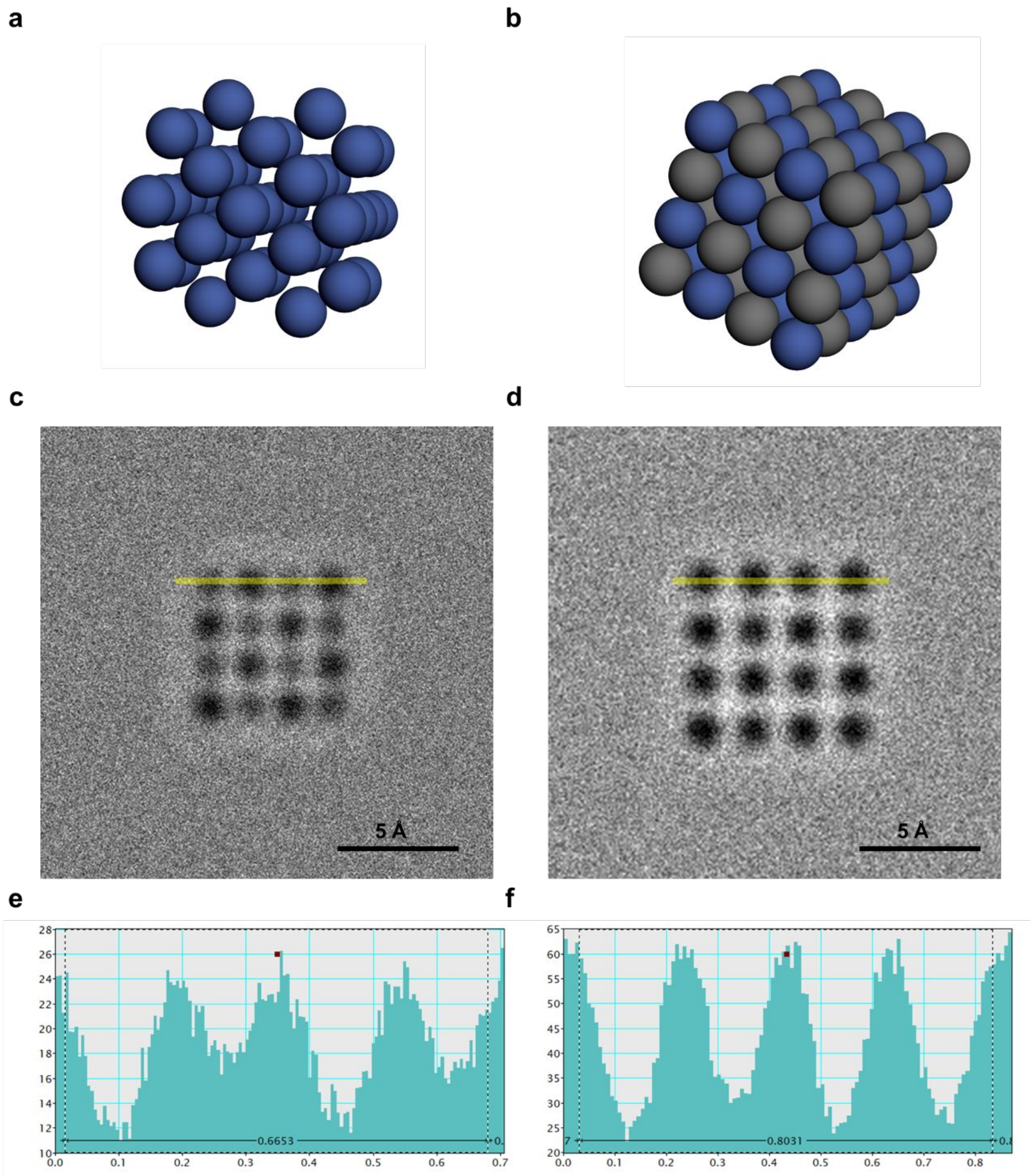

Figure S6. The size comparison between the Ni and NiC with the same structure. (a) 4×5×4 Ni model diagram. (b) 4×5×4 NiC model diagram. (c) QSTEM simulation image of the Ni in (a). (d) QSTEM simulation image of the NiC in (b). (e) Line profile of the yellow line in (c). (f) Line profile of the yellow line in (d).

By simulating the structure of pure nickel and nickel carbide, it is confirmed that carbon nanotubes can contain up to 4 layers of pure nickel atoms in the presence of van der Waals gaps. It is beneficial to determine the initial structure and the structure after full carbonization.

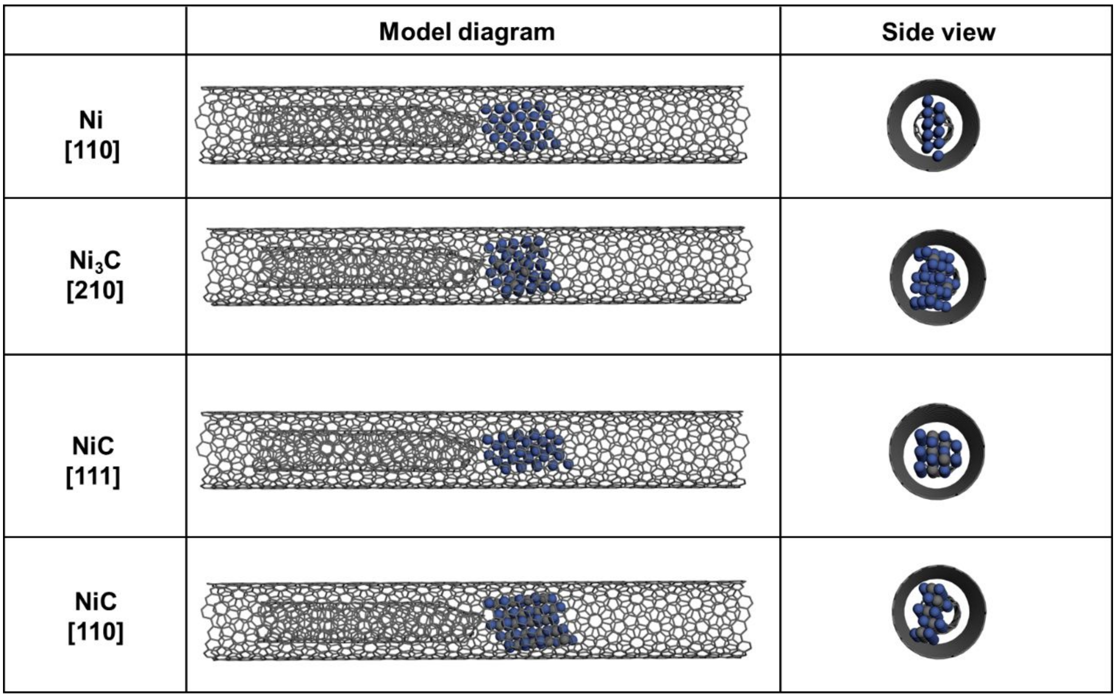

Figure S7. The different model diagram and side view of the 82 s image respectively are Ni [110], Ni_3C [210], NiC [111], NiC [110].

	Model diagram	Side view
**Ni 1layer**	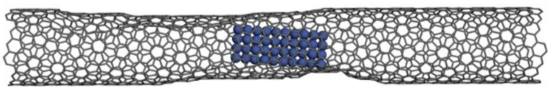	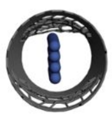
**Ni 2layers**	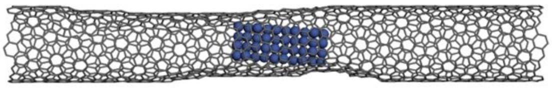	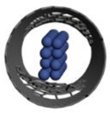
**NiC 2layers**	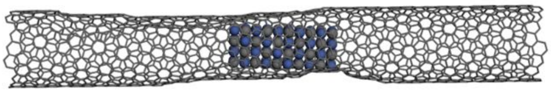	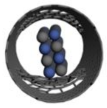

Figure S8. The different model diagrams and side view of the 207s image respectively are Ni-1L, Ni-2Ls, NiC-2Ls.

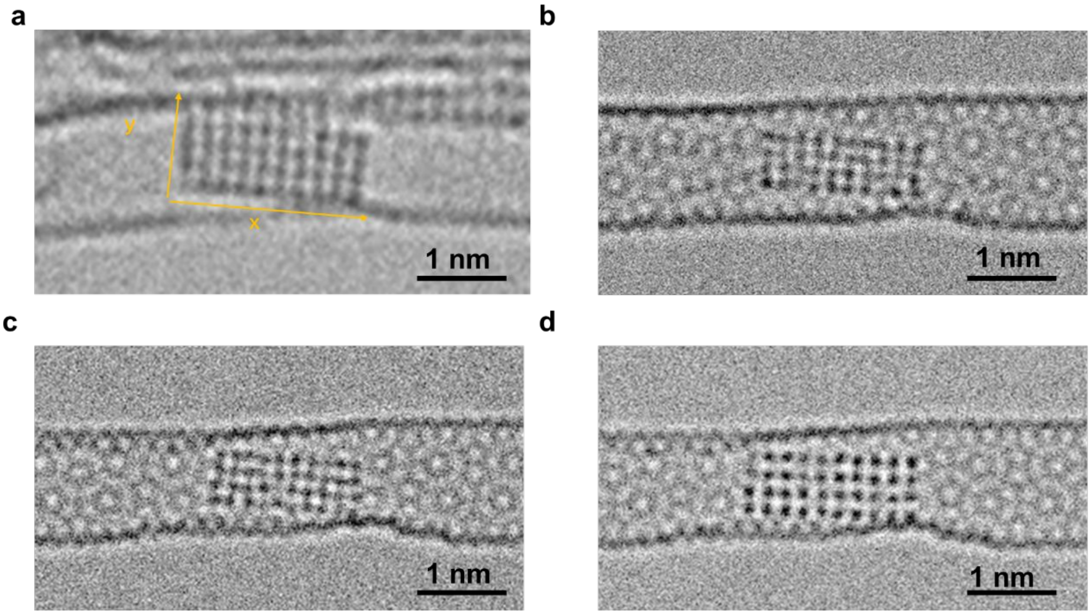

Figure S9. The raw AC-TEM(a) and corresponding simulation images of the three different models in Figure S6 respectively are Ni-1L(b), Ni-2Ls(c), NiC-2Ls(d).

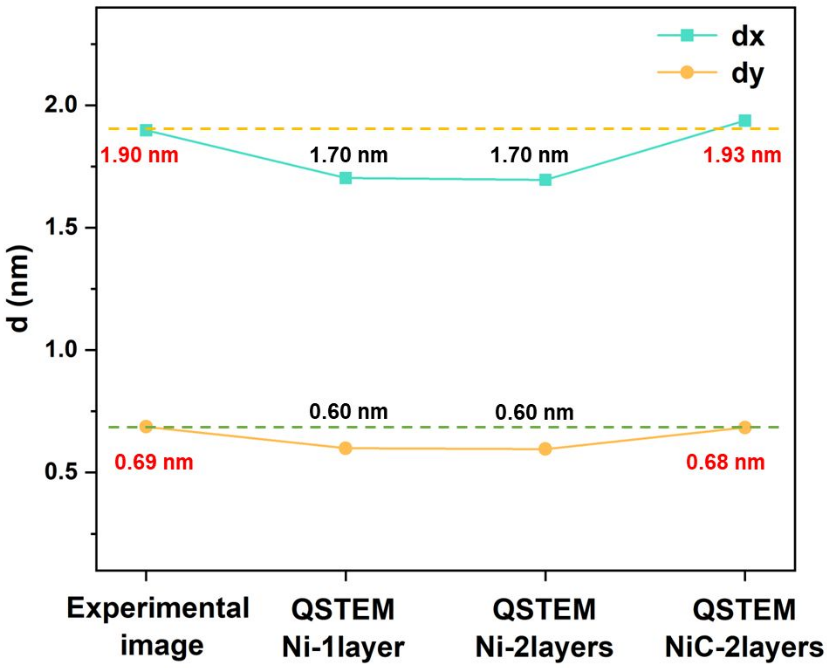

Figure S10. The length comparation between the raw image in 207s and different simulation images.

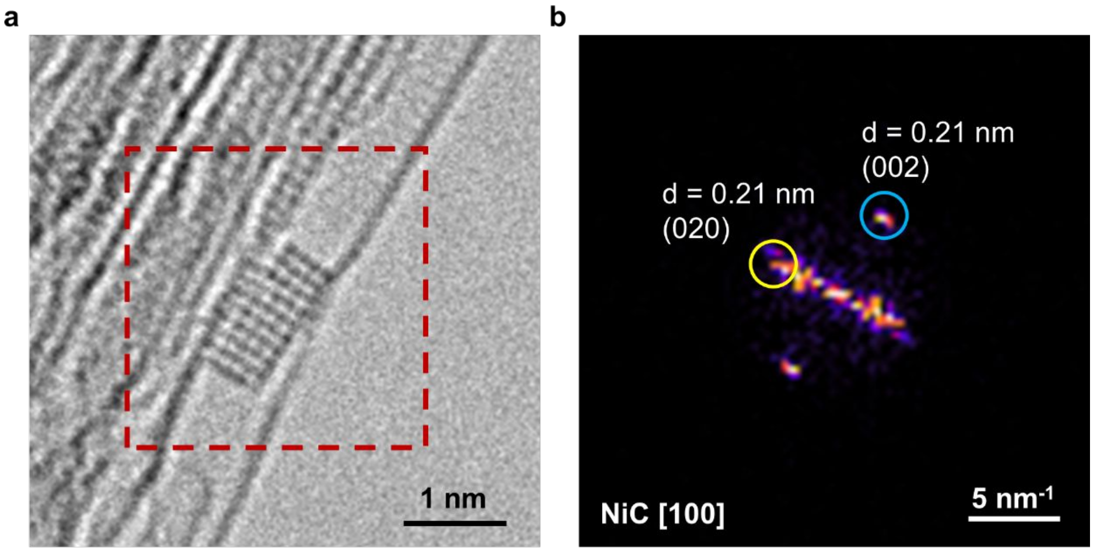

Figure S11. The FFT analysis of the fully carbonized metal nanocrystal in Figure 1. The FFT pattern shows a (002) group of crystal faces along the [100] zone axis.

We confirmed the symmetry information of the formed nanoparticles by FFT, and compare it with the standard structure of the suspected substance. We conduct FFT analysis on the two-dimensional structure formed at 207 s from Figure 1 in main text as illustrated in Figure S11. Considering its lattice shows cubic structure, we choose Ni and NiC structure for simulation and analysis. Through FFT analysis, we confirm the lattice distance matches NiC (002), proving NiC formed after the carbonization of nickel nanocrystals.

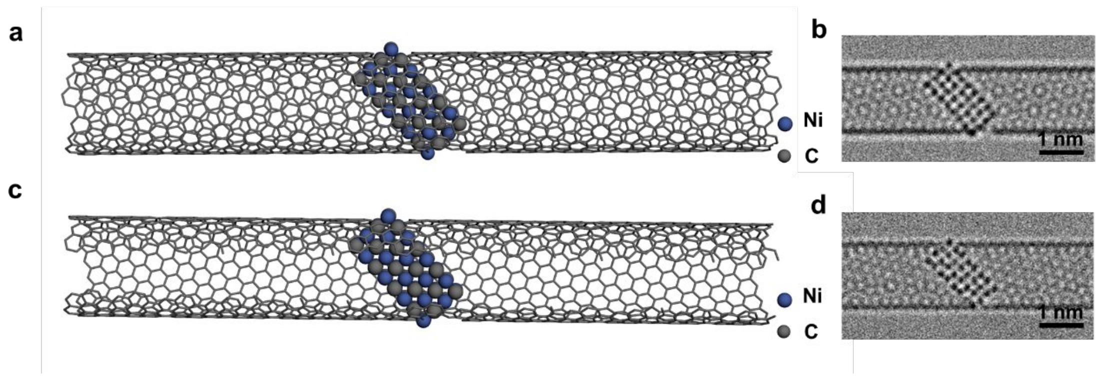

Figure S12. TEM simulation of the unbroken SWNT with NiC nanocrystal and the broken SWNT with nanocrystal. (a) The model diagram of the unbroken SWNT with NiC nanocrystal. (b) The corresponding TEM simulation image of (a). (c) The model diagram of the unbroken SWNT with the same NiC nanocrystal. (d) The corresponding TEM simulation image of (c).

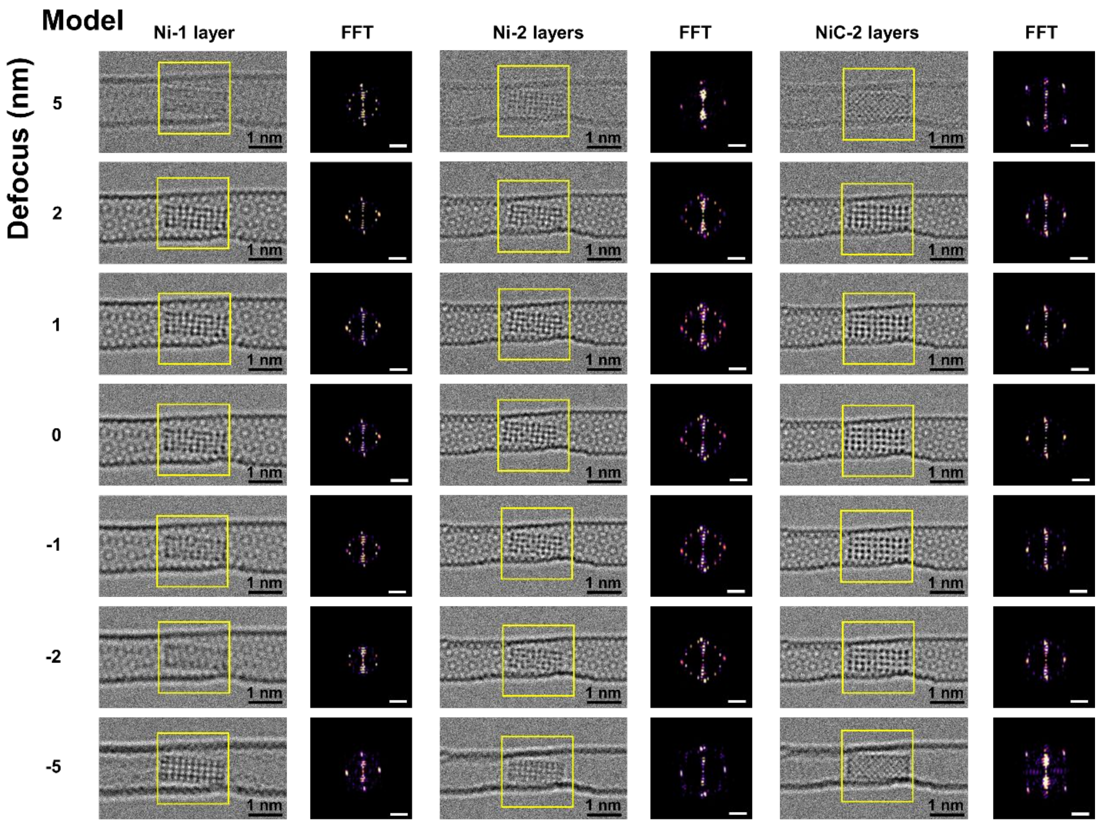

Figure S13. TEM simulation images with corresponding FFT patterns of the different models with defocus spread from -5 nm to 5 nm. The simulation results show the NiC structure matches perfectly to the experiment results. Scale bar in FFT patterns is 5 nm^{-1}.

Defocus is the most variable parameter during continuous TEM image capturing and recording, which is mainly caused by the vibration of the sample under electron beam irradiation. The change of defocus influences image quality and the reliability of image simulation. Other parameters, including astigmatism, coma and spherical aberration, do not change significantly during continuous capturing process lasting about 5 minutes. Therefore, we performed focus-spread TEM image simulations with varied defocus from -5 nm to 5 nm with corresponding FFT analysis as shown in Figure S13, proving that the symmetry information is affected even if the defocus changes during the experiments. The change of the defocus only influences the atomic contrast in simulated images.

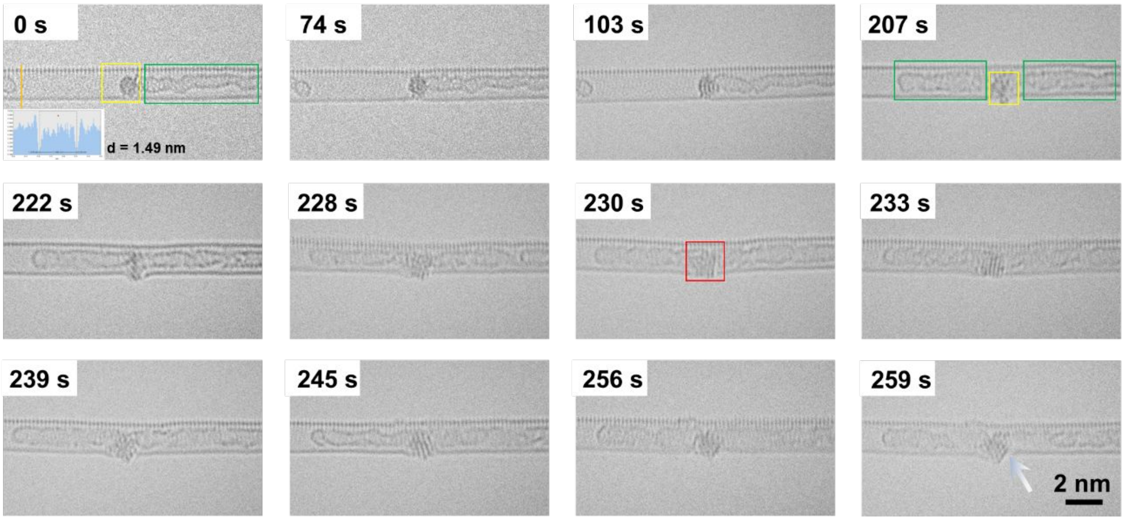

Figure S14. Carbonization process of a nickel nanocrystal confined in SWNT. The nickel nanocrystal (in yellow box) at 0 s connected with amorphous carbon (in green box) and the gradually carbonized structure at 230 s. At 259 s, the nanocrystal gradually cut the host SWNT. The blue arrow demonstrates the defect on SWNT. The diameter of SWNT is 1.49 nm.

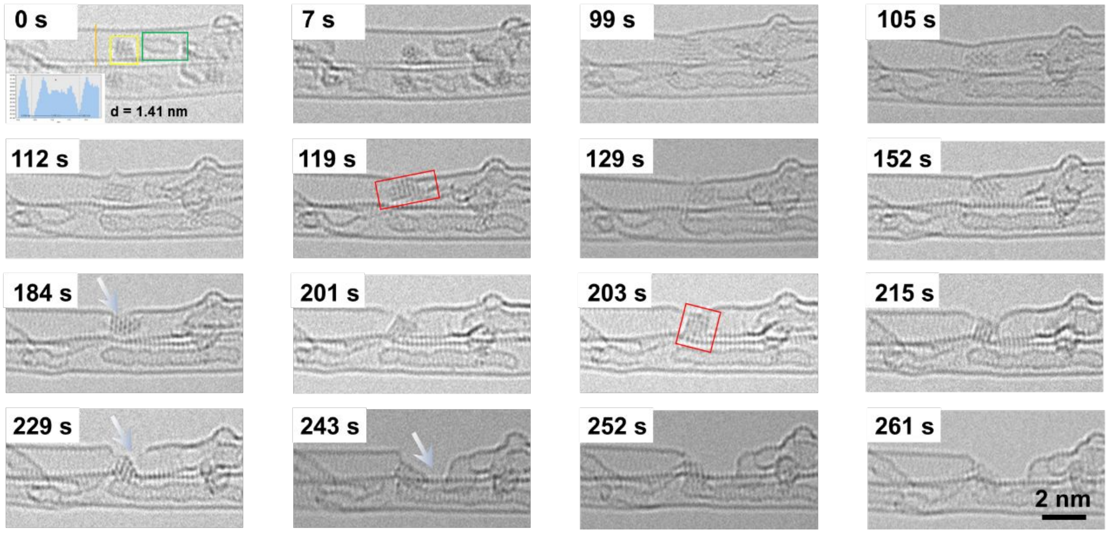

Figure S15. Time series AC-TEM images for the carbonization process of a nickel nanocrystal confined in SWNT. At 0 s, a nickel nanocrystal is indicated by yellow box with a neighboring amorphous carbon connected (in green box). Under 80 keV electron beam irradiation, this nickel nanocrystal gradually captured carbon atoms from amorphous carbon and the host SNWT, and carbonizes. At 119 s, the nanocrystal elongated structure and bonds to amorphous carbon and SWNT. At 203 s, the previous formed cubic NiC nanocrystal started cutting the host SWNT and created a big defect in the following 59 s. The diameter of SWNT is 1.41 nm.

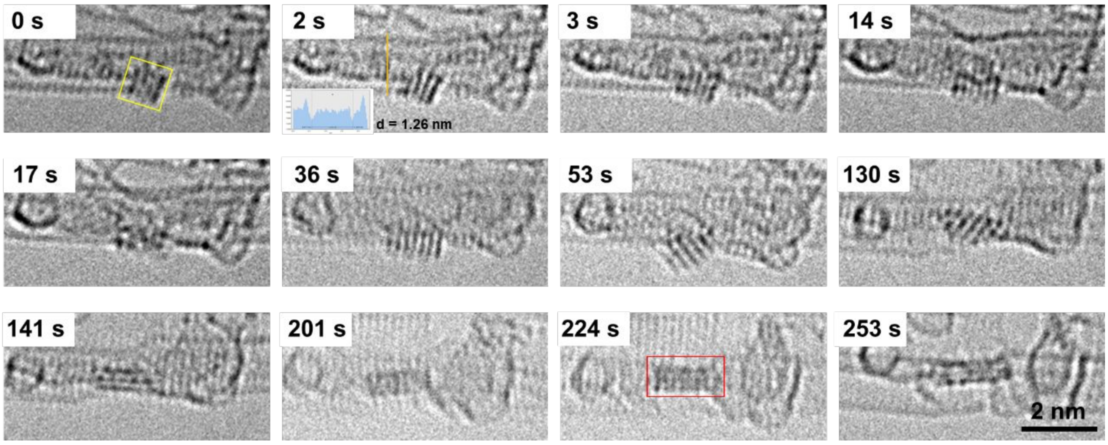

Figure S16. Time series AC-TEM images showing the carbonization process of a nickel nanocrystal. Similar to Figure 1 in main text, A cubic nickel nanocrystal (0 s, in yellow box) gradually carbonized to an elongated NiC nanocrystal (224 s, in red box) under electron beam irradiation. At 253 s, the already formed NiC nanocrystal cut the host SWNT and created a big defect. The diameter of SWNT is 1.26 nm.

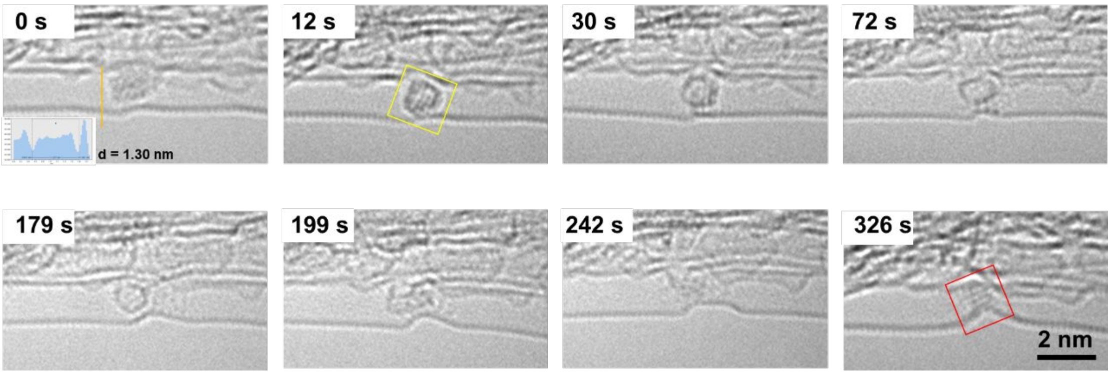

Figure S17. Time series AC-TEM images showing carbonization process of a nickel nanocrystal. A nickel nanocrystal (12 s, in yellow box) gradually carbonized to an NiC nanocrystal (326 s, in red box) under electron beam irradiation. The diameter of SWNT is 1.30 nm.

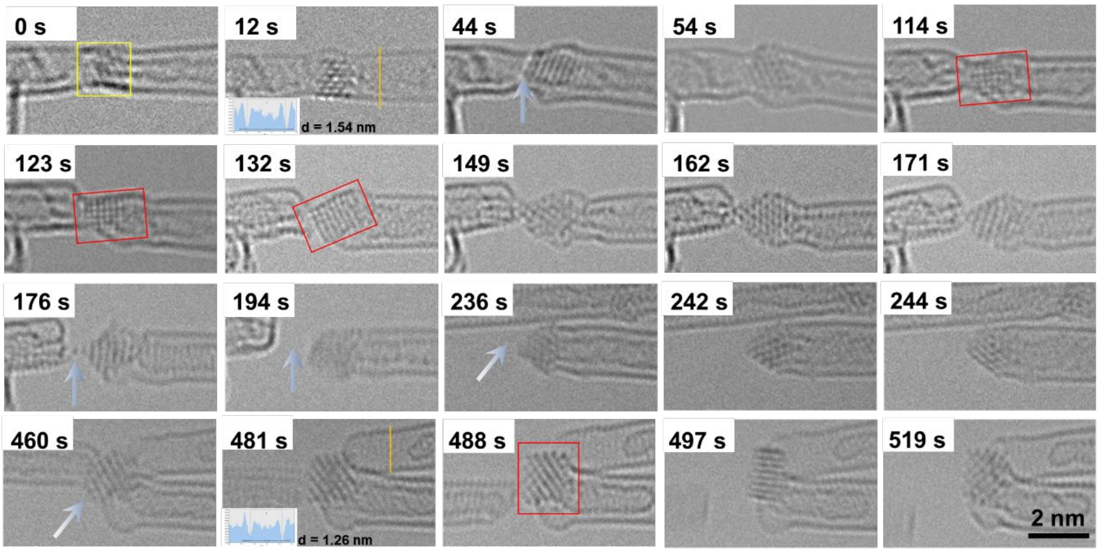

Figure S18. Time series AC-TEM images showing carbonization process of a nickel nanocrystal. At 0 s, a nickel nanocrystal (in fellow box) confined in the SWNT and bonded to amorphous carbon gradually carbonized to NiC nanocrystal. The reacting nanocrystal captured carbon atoms from the amorphous carbon and SWNT, resulting in the deformation of the host SWNT. From 114 s, the nanocrystal started cutting the SWNT. Interestingly, at 176 s, a Ni single atom was pulled out from NiC nanocrystal. And the NiC nanocrystal cut off the SWNT at 194 s. Consequently, the NiC nanocrystal swayed and attached to another SWNT. The diameters of SWNTs are 1.54 nm and 1.26 nm respectively.

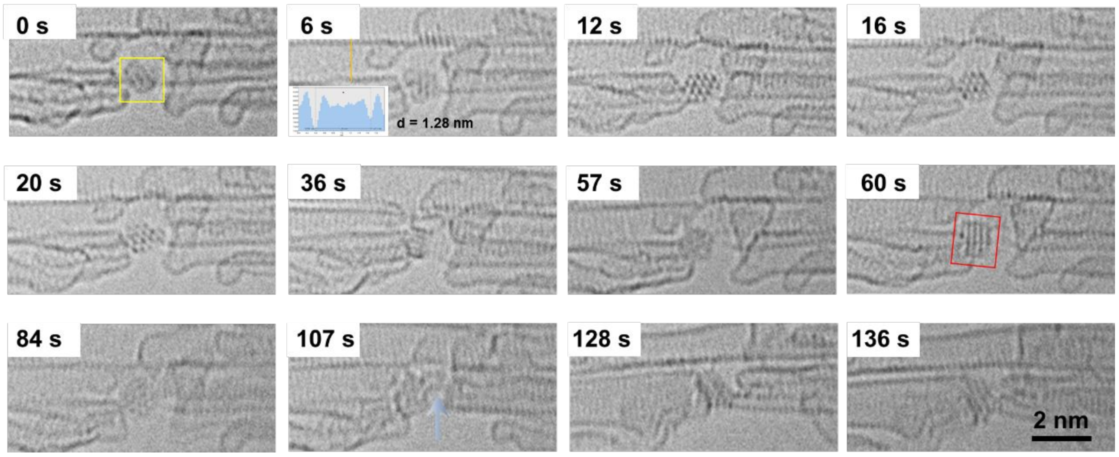

Figure S19. Time series AC-TEM images showing carbonization process of a nickel nanocrystal. Nickel nanocrystal in yellow box at 0 s was surrounded by amorphous carbon. It gradually carbonized under electron beam irradiation and cut off the SWNT, and eventually became NiC nanocrystal (in red box). The diameter of SWNT is 1.28 nm.

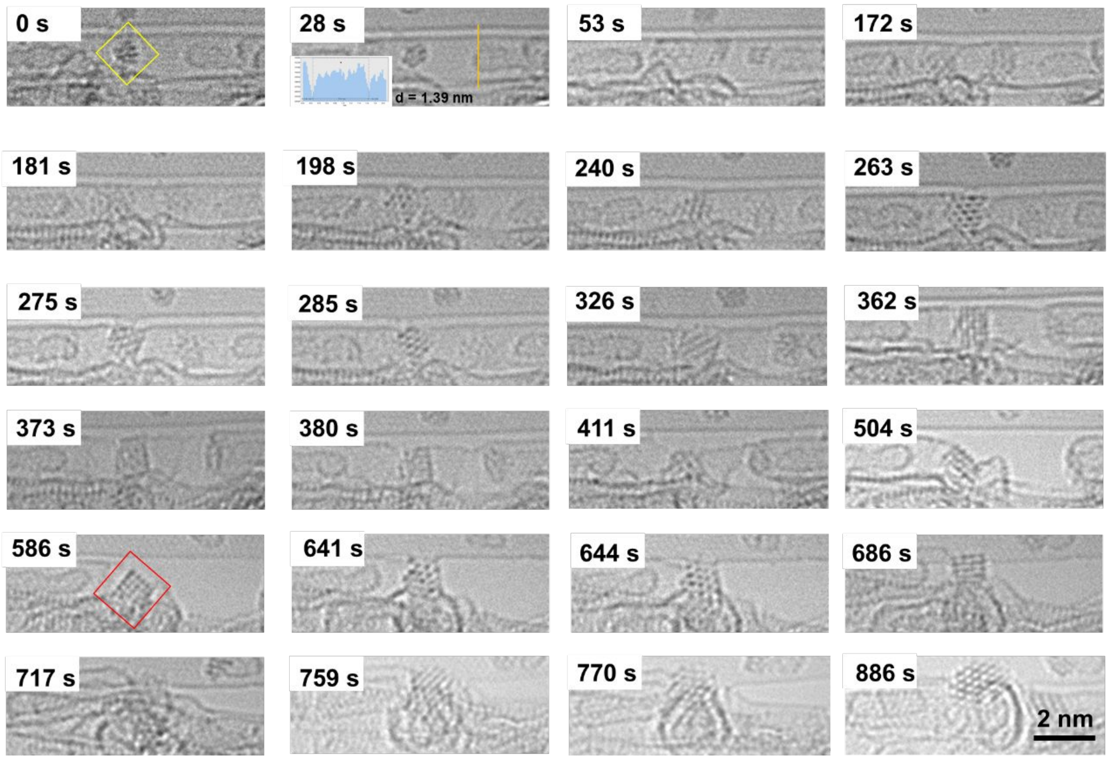

Figure S20. Time series AC-TEM images showing carbonization process of a nickel nanocrystal. Nickel nanocrystal at 0 s (in yellow box), gradually carbonized under electron beam irradiation and cut off the SWNT, and eventually became NiC nanocrystal (in red box). The diameter of SWNT is 1.39 nm.

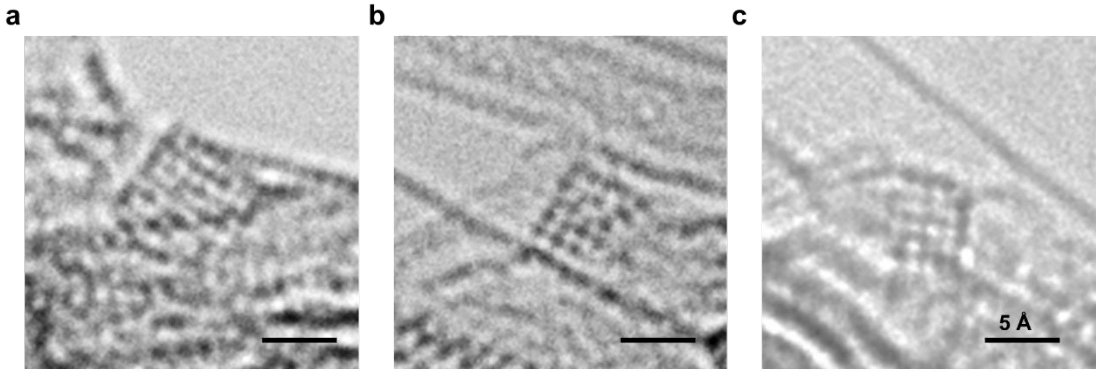

Figure S21. Formatted typical 2D NiC nanocrystals during our observation process.

Previous study indicates the metal carbide shows a 2D structure like LaC_2 confined in SWNT or AuC on graphene.[3-4]

Comparation of the thermally stimulated process and electron beam stimulated process

Based our previous study,[5] in AC-TEM experiments, energy is supplied directly to the atoms through kinetic energy transfer from the interaction between high energy electron beam and atoms. Under 80 keV electron beam irradiation, we found the different quasi-melt dynamic behavior of the metal nanoclusters.

According to the Gibbs-Thompson equation:[6]

$$\Delta T_m = T_m - T_m(d) = 4\sigma_{sl}T_m/(d\Delta H_f\rho_s)$$

the actual melting points for the nanometer scale nanocrystal will be several hundred degrees lower than for the bulk metal.

Where σ_{sl} is the surface energy of the solid-liquid interface, T_m is the bulk melting point, $T_m(d)$ is the melting point of crystals of size d, ΔH_f is the bulk enthalpy of fusion (per g of material), and ρ_s is the density of the solid.

We calculated the melting point of 1 nm nickel nanocrystal is 786.1 ℃ which indicates the relationship between the thermally activated reaction and the electron beam stimulated reaction in our experiments.

According the maximum energy transfer from the incident electron beam to atoms:[7]

$$E_{max} = \frac{2E_0(E_0 + 2m_0c^2)}{\mathrm{M}c^2}$$

80 keV incident electron beam transferred the maximum energy to nickel atoms is 3.23 eV. And we consider the cohesive energy of the nickel is 4.44 eV in analogy with the thermally stimulated process and electron beam stimulated process.

Where M = mass of atoms, c = speed of light, E_0 = energy of incident electron beam, m_0= mass of electron.

Previous studies have demonstrated that heat driven catalytic transformations of metals in carbon nanostructures, such as the Ni nanoparticle-catalyzed cutting of carbon nanotubes at 850°C[8] and the catalytic growth of carbon nanotubes by Ni, Co, and Re nanoparticles at various high temperatures exhibit similarities to the chemical reactions observed in TEM.[9-11] In our previous work,[5] we compared the melting points and cohesive energies of different transition metals, as well as the maximum kinetic energy transferred to metal atoms by an 80 kV electron beam. This comparison aimed to assess the extent to which chemical reactions induced by an 80 kV electron beam in carbon nanotubes resemble those driven by thermal energy in the range of 600-1000°C, even though our AC-HRTEM experiments were conducted at room temperature.

By exploring these phenomena at the nanoscale, we seek to extend traditional chemical concepts to a nano-meter level, enabling a deeper understanding of the underlying mechanisms.

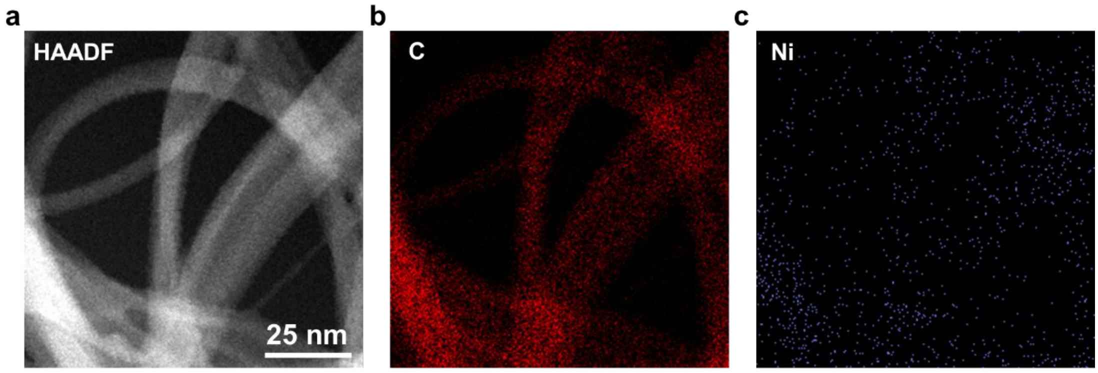

Figure S22. EDS mapping of the nickel nanocrystals confined in SWNT. (a) HAADF-STEM image of the nickel nanocrystals confined in SWNT. (b) Carbon element distribution in (a). (c) Nickel element distribution in (a).

Table. S1 The simulation parameters

Accelerate Voltage	80 keV
Simulation mode	TEM mode
Cs	0.015 mm
Coma	0 μm
3-fold astigmia	0 μm
Defocus	-9.7 nm (Scherzer mode)
Convergence angle	0.1mad
Tilt angle	Depends on the model

Video S1.

The carbonization of nickel nanocrystal confined in SWNT under electron beam irradiation (AVI).

Supporting references

(1) Cao, K.; Biskupek, J.; Stoppiello, C. T.; McSweeney, R. L.; Chamberlain, T. W.; Liu, Z.; Suenaga, K.; Skowron, S. T.; Besley, E.; Khlobystov, A. N. Atomic mechanism of metal crystal nucleus formation in a single-walled carbon nanotube. *Nat. Chem.* **2020**, *12* (10), 921-928.

(2) Jeon, S.; Heo, T.; Hwang, S.-Y.; Ciston, J.; Bustillo, K. C.; Reed, B. W.; Ham, J.; Kang, S.; Kim, S.; Lim, J. Reversible disorder-order transitions in atomic crystal nucleation. *Science* **2021**, *371* (6528), 498-503.

(3) Warner, J. H.; Ito, Y.; Rümmeli, M. H.; Büchner, B.; Shinohara, H.; Briggs, G. A. D. Capturing the motion of molecular nanomaterials encapsulated within carbon nanotubes with ultrahigh temporal resolution. *ACS Nano* **2009**, *3* (10), 3037-3044.

(4) Westenfelder, B.; Biskupek, J.; Meyer, J. C.; Kurasch, S.; Lin, X.; Scholz, F.; Gross, A.; Kaiser, U. Bottom-up formation of robust gold carbide. *Sci. Rep.* **2015**, *5*, 8891.

(5) Cao, K.; Zoberbier, T.; Biskupek, J.; Botos, A.; McSweeney, R. L.; Kurtoglu, A.; Stoppiello, C. T.; Markevich, A. V.; Besley, E.; Chamberlain, T. W. Comparison of atomic scale dynamics for the middle and late transition metal nanocatalysts. *Nat. Commun.* **2018**, *9* (1), 3382.

(6) Jackson, C. L.; McKenna, G. B. The melting behavior of organic materials confined in porous solids. *The Journal of Chemical Physics* **1990**, *93* (12), 9002-9011.

(7) Egerton, R. F.; McLeod, R.; Wang, F.; Malac, M. Basic questions related to electron-induced sputtering in the TEM. *Ultramicroscopy* **2010**, *110* (8), 991-997.

(8) Elías, A. L.; Botello-Méndez, A. R.; Meneses-Rodríguez, D.; Jehová González, V.; Ramírez-González, D.; Ci, L.; Muñoz-Sandoval, E.; Ajayan, P. M.; Terrones, H.; Terrones, M. Longitudinal Cutting of Pure and Doped Carbon Nanotubes to Form Graphitic Nanoribbons Using Metal Clusters as Nanoscalpels. *Nano Lett.* **2009**, *10* (2), 366-372.

(9) Ritschel, M.; Leonhardt, A.; Elefant, D.; Oswald, S.; Büchner, B. Rhenium-catalyzed growth carbon nanotubes. *J. Phys. Chem. C* **2007**, *111* (24), 8414-8417.

(10) Terrones, M.; Grobert, N.; Olivares, J.; Zhang, J. P.; Terrones, H.; Kordatos, K.; Hsu, W. K.; Hare, J. P.; Townsend, P. D.; Prassides, K.; et al. Controlled production of aligned-nanotube bundles. *Nature* **1997**, *388* (6637), 52-55.

(11) Yudasaka, M.; Kikuchi, R.; Matsui, T.; Ohki, Y.; Yoshimura, S.; Ota, E. Specific conditions for Ni catalyzed carbon nanotube growth by chemical vapor deposition. *Appl. Phys. Lett.* **1995**, *67* (17), 2477-2479.
